# Supplementary material for: Resolving the full spectrum of human genome variation using Linked-Reads
Source: Genome Res. 2019 Apr;29(4):635–45. doi: 10.1101/gr.234443.118 (PMC6442396; doi:10.1101/gr.234443.118)
Supplement: Supplemental Material [file supp_gr.234443.118_Supplemental_longranger-2.2.zip › longranger-2.2.tar.bz2!/lr2.2-public/lib/stan/cmdstan/stan/doc/api/html/arg__adapt__engaged_8hpp_source.html]

Stan: src/stan/gm/arguments/arg\_adapt\_engaged.hpp Source File


|  |  |
| --- | --- |
|  | Stan  2.5.0  probability, sampling & optimization |


- Main Page
- Related Pages
- Namespaces
- Classes
- Files

- File List
- File Members

All Classes Namespaces Files Functions Variables Typedefs Enumerations Enumerator Friends Macros Pages

- src
- stan
- gm
- arguments

arg\_adapt\_engaged.hpp

Go to the documentation of this file.

1 #ifndef STAN\_\_GM\_\_ARGUMENTS\_\_ADAPT\_\_ENGAGED\_\_HPP

2 #define STAN\_\_GM\_\_ARGUMENTS\_\_ADAPT\_\_ENGAGED\_\_HPP

3

4 #include <stan/gm/arguments/singleton\_argument.hpp>

5

6 namespace stan {

7

8  namespace gm {

9

10  class arg\_adapt\_engaged: public bool\_argument {

11

12  public:

13

14  arg\_adapt\_engaged(): bool\_argument() {

15  \_name = "engaged";

16  \_description = "Adaptation engaged?";

17  \_validity = "[0, 1]";

18  \_default = "1";

19  \_default\_value = true;

20  \_constrained = false;

21  \_good\_value = 1;

22  \_value = \_default\_value;

23  };

24

25  };

26

27  } // gm

28

29 } // stan

30

31 #endif

32

stan::gm::singleton\_argument::\_validity

std::string \_validity

**Definition:** singleton\_argument.hpp:151

stan::gm::arg\_adapt\_engaged::arg\_adapt\_engaged

arg\_adapt\_engaged()

**Definition:** arg\_adapt\_engaged.hpp:14

stan::gm::singleton\_argument::\_value

T \_value

**Definition:** singleton\_argument.hpp:154

stan::gm::valued\_argument::\_default

std::string \_default

**Definition:** valued\_argument.hpp:47

stan::gm::singleton\_argument

**Definition:** singleton\_argument.hpp:44

stan::gm::arg\_adapt\_engaged

**Definition:** arg\_adapt\_engaged.hpp:10

stan::gm::argument::\_name

std::string \_name

**Definition:** argument.hpp:76

stan::gm::singleton\_argument::\_constrained

bool \_constrained

**Definition:** singleton\_argument.hpp:157

stan::gm::singleton\_argument::\_default\_value

T \_default\_value

**Definition:** singleton\_argument.hpp:155

singleton\_argument.hpp

stan::gm::singleton\_argument::\_good\_value

T \_good\_value

**Definition:** singleton\_argument.hpp:159

stan::gm::argument::\_description

std::string \_description

**Definition:** argument.hpp:77


---

[ Stan Home Page ]

*© 2011–2014,
Stan Development Team.*
